# An Embryo State Training School

**Published:** 1896-11

**Authors:** 


### EDITORIAL DEPARTMENT.

F. E. DANIEL, M. D., Editor.
S. E. HUDSON, M. D., Managing Editor.
A. J. SMITH. M. D., Galveston, Associate Editor.

EDITORIAL STAFF:

PROF. J. E. THOMPSON, M. D., Texas Medical College, Galveston; Surgery.
PROF. WM. KEILLER, M. D., Texas Medical College, Galveston; Obstetrics and Gynecology.
PROF. DAVID CERNA, M. D., Texas Medical College, Galveston; Therapeutics.
PROF. A. J. SMITH, M. D., Texas Medical College, Galveston; Medicine
DR. R. H. L. BIBB, City of Mexico; Foreign Correspondent.

Published Monthly at Austin, Texas, by Drs. Daniel and Hudson. Subscription price $2.00 a year in advance.

Eastern Agents: The Monihan-Fairchild Special Agency, 24 Park Place, New York City.

Official organ of the West Texas Medical Association, the Houston District Medical Association, the Austin District Medical Society, Brazos Valley Medical Association, the Galveston County Medical Society, and several others

### AN EMBRYO STATE TRAINING SCHOOL.

Under the laws of Texas no physician is eligible to appointment to the position of Superintendent of either of the State lunatic asylums unless he has had actual experience in the management and treatment of the insane; (though we are sorry to say, in some instances, appointments have been made without regard to this requirement). There are three institutions of the kind in the State, with an aggregate capacity of some fifteen hundred; and every two years, i. e., with the change of administration, it becomes necessary to appoint a superintendent of each of these institutions, and two assistant physicians at each of two of them. The number of physicians in the State who are eligible to appointment under a proper construction of the law is limited to the ex-officers of those institutions, and can almost be counted on one's fingers. Hence the young men who have served as assistant physicians have a *cinch*, and are in demand; they become the superintendents, as a rule.

In this issue Dr. F. S. White, of Terrell, Texas, late superintendent of the State lunatic asylum at Austin, and who, perhaps, with one exception, has had a more extensive experience with the insane than any physician in the State, has a paper which contains a suggestion that might be adopted with benefit.

In each of the asylums there is an apothecary who receives a salary of $600 and found; and also a ward master or head nurse in each ward, who receives a salary of $300 and found. The doctor's suggestion is that these positions be filled by appointment—upon competitive examination, from the graduates of the Texas Medical College; that they be offered by the State as prizes.

This is an eminently sensible suggestion. In addition to the advantages pointed out by Dr. White, it would become in time a training school from which to draw the future officers of the institutions. These young men would in the course of their service qualify for appointment; they would in all probability succeed to the positions of first and second physician, and thus become eligible for appointment as superintendent. It would be a hot-bed, as it were, in which to sprout and propagate superintendents, and thus keep up a supply.

Under the law also—no physician is eligible to appointment as state health officer who has not had actual experience in the treatment of yellow fever; and as there have been no opportunities within recent years for the physicians of Texas to acquire such experience, the number who are eligible to hold the office under a strict construction of the law is reduced to a few individuals of the older set, who were called upon to encounter the dread pest away back in the sixties and seventies. Hence—in a few years—when these shall have passed away, it will be impossible to comply with the statute in regard to the experience feature—in making the appointment to this important office, and the law must of necessity be amended, repealed or disregarded. And yet it is hardly to be supposed that one totally inexperienced with yellow fever would want to hold the office—at the risk of encountering the disease, which he would surely do on shipboard some day. It is unfortunate, perhaps, that a remedy similar to the above cannot be found for this condition; it is hardly likely that a method could be adopted whereby one could qualify for State health officer by an experience and familiarity with yellow fever, unless it be possible through means of the quarantine service. Yellow fever comes to our doors almost every summer, but as a rule no one but the quarantine officer at Galveston sees it.

The JOURNAL hopes that Dr. White will bring this suggestion before the Board of University Regents, and that they will take steps to secure the legislation necessary to carry it into effect. It would serve as a stimulus to the young men studying medicine. Many of them, on graduating, have nothing but their diploma, and find it a difficult matter to set up in practice. Hence—while discharging light and not unpleasant duties—in the line of their chosen work, they would be at no expense for living, and the salary—as small as it is, could be saved up—the greater part of it; meantime they could prosecute their studies —or, if it is designed to train for the specialty of diseases of the mind and nervous system they would have exceptional advantages.

Apropos of Dr. White the JOURNAL learns and is pleased to announce that the committee to memorialize the legislature on relief for epileptics—in accordance with the doctor's views, as outlined in his paper on the subject, which appeared in our June, 1896, issue; and of which committee Dr. White is chairman—is actively at work in the line indicated, and will be prepared to bring the matter before the next legislature in a forcible manner.

Texas is away behind her sister States in many matters of the kind, and must catch up.

